# Living with Atypical Hemolytic Uremic Syndrome in the Netherlands: Patient and Family Perspective

**DOI:** 10.1016/j.ekir.2024.04.047

**Published:** 2024-04-27

**Authors:** Romy N. Bouwmeester, Leonie J. Engel, Wim Altena, Caroline Renette, Clim van Daelen, Evy van Kempen, Renée de Wildt, Nicole C.A.J. van de Kar

**Affiliations:** 1 Radboud University Medical Center, Amalia Children’s Hospital, Department of Pediatric Nephrology, Nijmegen, Netherlands; 2 Dutch Kidney Patients Association, Bussum, Netherlands; 3 The Netherlands Patients Federation, Utrecht, Netherlands

**Keywords:** aHUS, atypical hemolytic uremic syndrome, interviews, patient experiences, qualitative approaches, thrombotic microangiopathy

## Abstract

**Introduction:**

Atypical hemolytic uremic syndrome (aHUS) poses a significant health challenge due to its rarity and severity within the spectrum of thrombotic microangiopathy. Despite efforts to optimize and personalize health care for patients with aHUS, understanding the individual experiences, needs, and desires of patients with aHUS and their relatives remains limited.

**Methods:**

Here, we present a nationwide, exploratory, qualitative interview study with a direct content analysis approach. In-depth interviews and a 6-week evaluation were audio-recorded and conducted using a semistructured topic guide, based on the Institute for Positive Health (IPH) model.

**Results:**

Analysis of 10 interviews involving 6 patients with aHUS and 13 relatives revealed the prevalence of long-term disease symptoms in adult patients, notably fatigue, which significantly impacted daily functioning. Moreover, the resilience demonstrated by patients and their relatives was noteworthy; however, the acute phase of aHUS and the unpredictable nature of disease recurrence could profoundly affect mental well-being. The emotional toll of aHUS is pervasive, with feelings of fear, guilt, and trauma persisting across disease phases in both patients and relatives. Challenges in medical care, including delays in diagnosis and the need for personalized and uniform protocols, were highlighted. Support was deemed crucial, indicating the necessity for enhancements in the accessibility to comprehensible disease information and psychological counseling. Finally, complexities surrounding genetic testing and carriership were discussed.

**Conclusion:**

This study underscores the profound, enduring, and multifaced impact of aHUS. The insights gleaned from the experiences and needs of patients with aHUS and their relatives could lay the foundation for development and implementation of more personalized innovations in aHUS health care.

## Graphical abstract

**Figure:**
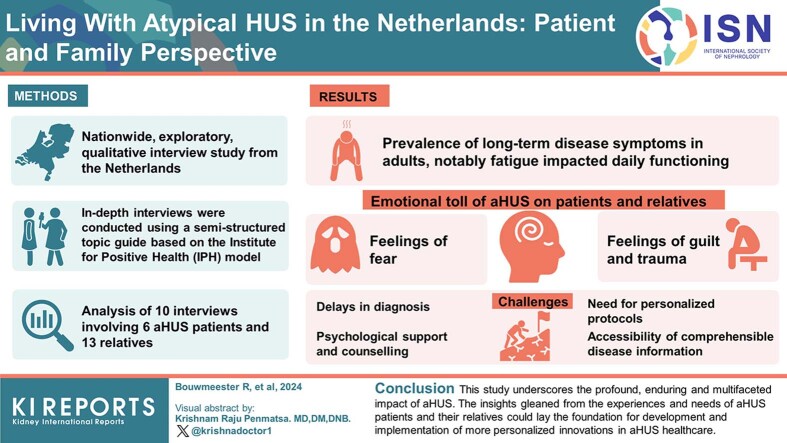

Atypical HUS is a rare and severe form of a thrombotic microangiopathy, characterized by hemolytic anemia, thrombocytopenia, and acute kidney injury.^1^ Atypical HUS affects 5 to 10 individuals per year in the Netherlands (estimated prevalence 120–150/17,500,000), can occur at any age, and is the result of an uncontrolled overactivation of the complement system, usually following a triggering event (e.g., infection). Diagnosing aHUS is complicated, which often contributes to a delay in time and insecurity in some patients. This disease is known for its potentially relapsing character and (likely) pathogenic variant(s) in complement proteins can be identified in up to 70% of patients with aHUS.^2^, ^3^, ^4^ Genetic variant carriership can also be found in family members; however penetrance is incomplete and the risk of disease onset is relatively low (9%–19%).^5^ Historically, plasma therapy was the only treatment option for aHUS, yet was associated with poor outcome perspectives, including high mortality rates (2%–10% in the acute phase) and end-stage kidney disease in up to 50% of patients.^6^, ^7^, ^8^

In 2011, a revolutionary new treatment option was introduced: eculizumab.^9^ This marked the beginning of a new era for both treatment and outcomes of patients with aHUS. Although eculizumab greatly improves the health-related quality of life, treatment with eculizumab is not without risks, including an increased susceptibility to meningococcal infection.^10^ With costs for eculizumab of up to €500,000 per patient with aHUS per year, cost-effectiveness is lacking. Since 2016, Dutch patients with aHUS are treated following a restrictive eculizumab regimen which was recently confirmed to be safe, without negatively influencing quality of life.^4^ However, considering the risk of relapse(s), close monitoring remains mandatory and patients are instructed to, at all times, be aware of potential disease triggering factors. Consequently, physicians, patients, and relatives share responsibility in disease monitoring.

Although the scientific understanding of biochemical mechanisms of aHUS and therapeutic options are continuously increasing, little is known about living with this life-changing diagnosis and its potentially relapsing character. It is time to again embark on a new era of aHUS health care; 1 that prioritizes the patient as a whole, not just their disease. This study aims to evaluate the personal experiences, needs, and desires of Dutch patients with aHUS and their relatives through exploratory, in-depth interviews using the broad perspective of the positive health model as a guide.

## Methods

### Design

This is an exploratory, qualitative interview study with a direct content analysis approach. The end point was the reach of theoretical data saturation. All interviews were conducted from August 2020 until April 2021, directly following a nonrecorded pilot phase. Findings were reported following the Consolidated Criteria for Reporting Qualitative Studies checklist (Supplementary Table S1).^11^ Ethical approval was obtained from the Medical Research Ethics Committee of Oost-Nederland (Eigen Regie, 2019-6008).

### Participants Selection

This study was a collaboration between the Dutch Kidney Patients Association and the Department of Pediatric Nephrology, Radboud University Medical Center, Nijmegen, the Netherlands, acknowledged as European Rare Kidney Disease Reference Center. Patients and relatives were recruited via the Dutch Kidney Patients Association (social media) and/or their physician (face-to-face), initially using the consecutive and snowball sampling approach. Research objectives were shared both verbally and in text during recruitment. After inclusion of 3 adult patients (of whom 1 was a kidney transplant recipient), 4 parents and 1 spouse, 3 participants (1 additional kidney transplant recipient, 1 spouse, and 1 sibling) were purposively recruited to ensure as much variety in participant demographics as possible. The remaining participants were recruited using the original consecutive approach.

Initially, only Dutch speaking patients with aHUS (aged >15 years), their first-degree relatives, and spouses were considered eligible to participate. One-to-one interviews were pursued. However, if explicitly requested by the participant, children (aged ≤15 years) were granted participation under the supervision of their parent(s) and/or interviews with multiple family members could be conducted at once, conditionally upon a comfortable, nonjudgmental environment. Participation of family members could be independent from inclusion of the related patient. All participants gave informed consent before inclusion in the study.

### Data Collection

The aim of this study corresponds to the broad perception of health, defined as the ability to adapt and self-manage in the face of social, physical, and emotional challenges. Interviews were semi structured using the IPH as part of the topic guide.^12^ This model enables a more personalized evaluation of the situation and contains 6 dimensions of health on a subjective scale, visualized in a spiderweb-diagram for practical use. Implementation of this model has shown to improve communication, focusing on questions such as: “What really matters to you?” and “What would you like to change?” At the start of the interview, interviewers explained the IPH model after which participants filled in the diagram for their current situation and choose domains to discuss further.

Three interviewers were trained in aHUS disease characteristics and the IPH model. Interviewers were provided an extensive topic guide form (Supplementary Material: Topic Guide Interview and Evaluation). All interviewers had experience in coaching and counseling and were (intentionally) not involved in the health care of the participants. Interviews were held at a place chosen by the interviewee; however, due to COVID-19 restrictions, several interviews had to be conducted online using Zaurus (version 1.0.3.273). Approximately 6 weeks after the initial interview, a 10 to 20-minute telephone evaluation with the same interviewer was conducted to enable reflection on the emotional impact of the interview and provide feedback on the perspective summary. All interviews and telephone evaluations were audio-recorded and analyzed uniformly.

### Data Analysis

The interviews were transcribed verbatim and anonymized. Conventional thematic content analysis was performed. The first 4 interviews were independently and openly coded line-by-line by 2 researchers (RNB and LJE), after which a consensus meeting was held to develop a codebook. Due to sufficient similarity in coding, the remaining interviews were initially coded by 1 researcher (LJE) according to this codebook, but critically reviewed and coded axially by the other researcher (RNB). ATLAS.ti Web (version 22.0.11) was used to support this process. Finally, categories were selectively coded into themes. Categories, themes, and data saturation were regularly discussed with the entire research team. This team consisted of a pediatric nephrologist (NCAJvdK), a medical doctor/PhD candidate trained in qualitative research (RNB), biomedical science student (LJE), and 5 members of the Dutch Kidney Patients Association: 2 patient advocates (WA, CR: also, interviewer) and 3 policy-officers (EvK, RdW, and CvD). Although data was initially coded using an inductive approach, final themes could also be deductively matched with several IPH-model categories. Completed IPH spiderweb-diagrams were evaluated to help establish the most relevant themes. Visual representations of the diagrams were not included here because these snapshots were not considered a good representation of the definite needs and desires. Observation notes of the interviews were obtained. Thereby, essential information regarding, for example, nonverbal communication could be included in the data analysis. Quotes that best reflected a theme were selected. All quotes were evaluated on full preservation of anonymity of the participant and accompanied by a letter (Patients: P,^1^, ^2^, ^3^, ^4^, ^5^, ^6^ Relatives: R^1^, ^2^, ^3^, ^4^, ^5^, ^6^, ^7^, ^8^, ^9^, ^10^, ^11^, ^12^, ^13^).

## Results

Thirteen interviews with a total of 7 patients with aHUS and 15 relatives were conducted. Unfortunately, 3 online interviews (1 patient and 2 relatives) were excluded from further analysis due to insufficient quality of the audio recordings. Characteristics of all remaining interviewees are shown in Table 1. The interviews lasted between 60 and 90 minutes. The final codebook consisted of 928 codes, which were merged into 39 categories and 11 major and 2 minor themes (Table 2).

**Table 1:** Participant characteristics

Characteristic	Patients (*n* = 6)	Relatives (*n* = 13)	Participants total (*N* = 19)	Individual participants codes
Sex, male, *n* (%)	5 (83%)	4 (31%)	9 (47%)	
Median age at interview, yr (min-max)	52 (11–66)	45 (19–65)	48.5 (11–66)	
Medical history, *n* (%)				
aHUS	6 (100%)	0 (0%)	6 (32%)	P^1^, P^2^, P^3^, P^4^, P^5^, P^6^
aHUS genetic variant (carrier)	6 (100%)	1 (8%)	7 (37%)	P^1^, P^2^, P^3^, P^4^, P^5^, P^6^, R^10^
Kidney transplantation	2 (33%)	4^a^ (31%)	6 (32%)	P^1^, P^4^, R^2^, R^3^, R^10^, R^11^
Current treatment*, n* (%)				
Eculizumab continuous treatment	0 (0%)	0^a^ (0%)	0 (0%)	
Eculizumab discontinued	1 (17%)	7^a^ (54%)	8 (42%)	P^2^, R^1^, R^4^, R^5^, R,^6,7^ R^8^, R^12^
Relation, *n* (%)				
Patient	6 (100%)		6 (32%)	P^1^, P^2^, P^3^, P^4^, P^5^, P^6^
Parent		6 (46%)	6 (32%)	R^1^, R^4^, R^5^, R^8^ , R^9^, R^13^
Spouse		2 (15%)	2 (11%)	R^2^, R^3^
Child		1 (8%)	1 (5%)	R^10^
Sibling		1 (8%)	1 (5%)	R^11^
Grandparent		2 (15%)	2 (11%)	R^6,7^
Aunt		1 (8%)	1 (5%)	R^12^

Table 1 provides an overview of participant characteristics. Kidney transplantation and current treatment of relatives represents the history and treatment of the related patient and not the relative.

aHUS, atypical hemolytic uremic syndrome.

a In (related) aHUS patient(s), not relative(s).

**Table 2:** Overview of themes and categories

Major themes	Categories	Participant coverage
Bodily functions	Acute phase symptoms	R,^1^, ^2^, ^3^, ^4^, ^5^, ^6^, ^7^, ^8^, ^9^, ^10^, ^11^ P^1^, ^2^, ^3^, ^4^
	Long-term symptoms	R,^1^, ^2^, ^3^, ^4^, ^5^, ^6^, ^7^, ^8^, ^9^ R^11^, P^1–4^
	No long-term symptoms	R^1^, R,^4^^,^^5^ R,^8^^,^^9^ R^11^, P^2^, P^4^
	CKD (related)	R,^2^, ^3^, ^4^, ^5^ R^8^, R^11^, P^1^, ^2^, ^3^, ^4^
	Fatigue	R,^6^^,^^7^ R^9^, P,^1^^,^^2^ P^4^
	Other	P^2^^,^^3^
	Relapse	P^1^
	Other	R^2^, R^5^, R^8^, P^1^
Daily functioning	Limitations	R,^1^, ^2^, ^3^, ^4^ R^11^, P^1–4^
	Impact on mental well-being	R,^1^, ^2^, ^3^, ^4^ R^11^, P^1–4^
	Lifestyle/participation changes	R,^1^, ^2^, ^3^ R^11^, P^2^
	General	R,^1^, ^2^, ^3^ P^1^, ^2^, ^3^
	No limitations	R,^1^^,^^2^ R,^4^^,^^5^ R,^8^^,^^9^ P^1^, ^2^, ^3^
	Participation	R,^1^, ^2^, ^3^, ^4^, ^5^, ^6^, ^7^, ^8^, ^9^ R^12^, P^1–4^
Mental well-being		
Acceptance and meaningfulness	General	R,^1^^,^^2^ R^4^, R,^6^^,^^7^ R^10^, R^12^, P^1^, ^2^, ^3^
Navigating uncertainty and seeking empowerment	General	R,^1^, ^2^, ^3^, ^4^ P^2^
Persistent emotional impact		
	Emotional impact	R,^1^, ^2^, ^3^, ^4^, ^5^, ^6^, ^7^, ^8^, ^9^ R,^11^^,^^12^ P^1^, ^2^, ^3^, ^4^
Impact of relapse	No emotional impact	R^11^, P^1^, P^2^
	Relapse	R,^1^, ^2^, ^3^, ^4^, ^5^, ^6^, ^7^, ^8^, ^9^ R^12^, P^1–3^
	Potential impact	R,^1^, ^2^, ^3^, ^4^, ^5^, ^6^, ^7^, ^8^, ^9^ R^12^, P^1–3^
Impact of eculizumab discontinuation	Other	P^1^
	Eculizumab discontinuation	R^1^, R,^4^, ^5^, ^6^, ^7^, ^8^ P^2^, R^12^
	General	R,^1^, ^2^, ^3^, ^4^, ^5^, ^6^, ^7^, ^8^, ^9^ R^12^, P^3^
	Quality of life	R,^1^^,^^2^ R,^4^^,^^5^ R,^8^, ^9^, ^10^, ^11^ P^2^, P^4^
	Meaningfulness	R^1^, R,^9^, ^10^, ^11^ P^1^, P^3^
Finding support and overcoming isolation	Lack of support	R,^1^, ^2^, ^3^, ^4^, ^5^, ^6^, ^7^, ^8^, ^9^, ^10^, ^11^, ^12^ P^1^, ^2^, ^3^, ^4^
	General (incl. isolation)	R,^1^, ^2^, ^3^ R^5^, R,^8^^,^^9^ P^3^
	Professional support	R,^2^, ^3^, ^4^, ^5^, ^6^, ^7^, ^8^ R^12^, P,^1^^,^^2^ P^4^
	(Peer) support system	R,^1^, ^2^, ^3^, ^4^, ^5^, ^6^, ^7^, ^8^, ^9^, ^10^, ^11^, ^12^ P^1^, ^2^, ^3^, ^4^
	Other	R^1^, R,^4^^,^^5^ R,^8^^,^^9^ P^1^, P^3,4^
Information accessibility and clarity	Lack of information	R,^1^, ^2^, ^3^, ^4^, ^5^, ^6^, ^7^, ^8^, ^9^, ^10^, ^11^, ^12^ P^1^, P^4^
	Information (source) desires	R,^1^, ^2^, ^3^, ^4^, ^5^, ^6^, ^7^, ^8^, ^9^, ^10^, ^11^, ^12^ P^1^, P^3,4^
	Other	R^1^, R^3^, R^5^, R,^8^^,^^9^ R^11^, P^1,2^
Challenges in medical care	General	R,^1^, ^2^, ^3^, ^4^, ^5^, ^6^, ^7^, ^8^, ^9^ R,^11^^,^^12^ P^1^, ^2^, ^3^, ^4^
	Acute phase	R,^1^, ^2^, ^3^, ^4^, ^5^, ^6^, ^7^, ^8^, ^9^ R,^11^^,^^12^ P^1^, ^2^, ^3^, ^4^
	Convalescent phase	P^1^
	Selfcare	R^1^, R^4^, R,^6^^,^^7^ R^12^, P^1–3^
	Other	R^1^
	Positive experiences	R,^1^^,^^2^ R,^4^, ^5^, ^6^, ^7^, ^8^, ^9^, ^10^ R^12^, P^1–4^
	Negative experiences	R,^1^, ^2^, ^3^, ^4^, ^5^, ^6^, ^7^, ^8^, ^9^ R^12^, P^1–4^
	Needs or desires	R,^1^, ^2^, ^3^, ^4^, ^5^, ^6^, ^7^, ^8^, ^9^, ^10^ R^12^, P^1–4^
Carriership	Genetic testing Physical and Mental impact	R^2^, R^4^, R,^6^^,^^7^ R,^11^^,^^12^ P^1^, P^4^ R,^2^, ^3^, ^4^ R,^6^^,^^7^ R,^10^, ^11^, ^12^ P^1^, P^4^
Minor themes		
COVID-19 pandemic	COVID-19 pandemic	R^3^, P,^3^^,^^4^ R^1^, R^5^, R^8^, R^11^
Transplantation^a^	Bodily functions	R,^2^, ^3^, ^4^, ^5^ R^8^, R^11^, P^1^, P^3,4^

CKD, chronic kidney disease.

a Data too patient-specific and therefore not included in this article.

### Long-Term Symptoms and Daily Life Impact

Parents commonly reported that none to only minor long-term symptoms were present that could hinder bodily or daily functioning of their child. In the acute phase, children did miss school and hobbies. However, as they regained physical strength, these activities could be rapidly resumed. One parent (R^1^) mentioned: “Actually there were 2 very intense weeks (in the acute phase), but afterwards, it aHUS was under control, and it only got better and better.” On the contrary, nearly all adult patients expressed a persistent presence of fatigue, despite differences in disease severity during the acute phase (e.g., need for dialysis or transplantation). If present, long-term symptoms evidently restricted daily functioning, especially the ability to fulfill paid work: “But then, slowly, we came to the conclusion that working might become a problem. That is what I struggled with the most” (P^2^). However, participation in volunteering work and/or patient advocacy did bring a new dimension of meaningfulness to some participants.

Limitations in daily functioning did not affect patients only, but also extended to their relatives who had to adapt to a new, sometimes permanent, situation that necessitated changes in family lifestyle, including forgoing (specific) holidays or spontaneous activities. “I have been so angry. Like, I couldn’t travel anymore, and I was stuck with someone sick. I didn’t sign up for that” (R^3^).

### Persistent Emotional Impact

Regardless of the disease phase, aHUS had a persistent impact on both patients and relatives’ lives. Or, as a spouse (R^3^) illustrated: “His illness is always present in the background. Even though he is doing okay now. It is just always there. It is never gone.” For the majority of patients with aHUS, their mental well-being was often acknowledged, especially during the acute phase. However, the emotional toll (which included feelings of fear and guilt) on their relatives was generally overlooked. Two relatives reported: “As a spouse you also have to deal with the fear of losing your partner”(R^3^) and “Now I know that there is nothing I could have done differently or sooner, but in the beginning, I just had a very strong feeling that I did something wrong” (R^4^). One spouse (R^2^) shared her trauma: “For heaven’s sake, let nothing happen that would make me have to go through another trajectory again. That I would have to suffer again and go through all those hospital visits once more.” To some patients, the emotional impact on their relatives would even outweigh their own: “That (my kids would worry about me) is hard. I find it even harder than being sick myself” (P^2^).

#### Survival Mode

During the acute phase, participants often reported being in survival mode. Particularly, spouses and parents were forced to deal with the emotional impact for an extended period of time: “Children have seen unforeseeable things, but especially parents and spouses were forced to be in survival mode for a long period of time” (P^1^). One relative (R^2^) adds: “When the acute phase is over, it (fear) dampens a bit, but there is always a sort of ‘undercurrent’ from which you keep having the fear of ending up in it (aHUS episode) again.” Moreover, once out of survival mode, the impact could become fully apparent: “When it went better with (name patient) I had a severe burnout. Of course, just then it finally hit me. And it hit me long”(R^3^).

### Impact of Eculizumab Discontinuation and Relapse

For some participants, the emotional impact of aHUS was fueled by the fear of relapse, particularly during potentially triggering events, which was even more pronounced during the COVID-19 pandemic. However, discontinuing eculizumab itself was not mentioned as severely impacting quality of life. A parent (R^1^) stated: “The most problematic is whether we will be on time (in case of a relapse). However, it is so reassuring that if it (eculizumab in case of disease recurrence) is needed, she (patient) can receive it within 1 hour.” Participants mentioned that over time, after multiple potentially triggering events, they obtained a sense of confidence: “It's been years- I think this is the seventh or sixth year that it's been going really well, and we haven't had a relapse. At some point you will gain confidence in her body again” (R^5^) and “Every time I thought it (aHUS) was coming back, I was really kind of like scared actually. And it was scarier back then than it is now basically. I have had multiple infections, so now I'm a little less worried” (P^3^). Finally, advanced perception of their disease was found to reduce distress: “I’m really relieved that I know where (alarm symptoms) to look out for” (R^3^).

### Navigating Uncertainty and Seeking Empowerment

At times, participants struggled with the need for alertness, due to the unpredictability of aHUS: “If he (patient) gets sick or whatever, that is when my alertness starts” (R^8^). One patient (P^2^) adds: “The inability to control the situation, that was for me personally, the most difficult.” Some participants have actively searched for ways to gain more control over their situation. A parent (R^1^) mentioned that: “I searched pretty hard for what would give me more certainty.” Overall, better understanding of aHUS was considered contributory to their sense of empowerment (see Information Accessibility): “It would put my mind at ease knowing that I had a little bit more ammunition (knowledge). That I will be able to say the right things to a doctor, what will trigger him/her to take me seriously” (R^2^).

### Acceptance and Meaningfulness

Although the path and pace toward acceptance varied, it was consistently cited as a significant topic, and often included processing a (potentially) traumatizing event and physical restrictions. There were some factors that were attributed to acceptance, including the reassurance “that more aHUS patients suffer from this (long-term symptoms)” (P^3^). Another patient (P^1^) pointed out: “I think quality of life is also a bit whether you accept the situation you find yourself in. It is just that. And within the current limitations of what we can do or where we can go, it’s a really nice life.” A spouse (R^2^) mentioned that despite the physical complaints of her partner, they remained optimistic: “It is what it is. Luckily, we are still able to do a lot of things.” In fact, almost all patients with aHUS and their relatives eventually found a way to even see a positive side to their disease, as illustrated by the saying of 1 patient (P^3^): “It (aHUS onset) was such a turning point in my life. In fact, I have only started to see life more positively because of my illness. Because of what happened to me, there is some kind of extra appreciation to life. The first thing I really noticed is how many caring people I have around me, something you do not necessarily notice within day-to-day life.”

### Finding Support and Overcoming Isolation

Participants emphasized support from family and friends as an important factor: “I think that (my support system) is what makes my happiness for a big part” (P^3^) and “we really appreciated that people just came by (in the acute phase) and support you” (P^4^). Nevertheless, some did experience feelings of isolation and loneliness, often due to the severity and/or complexity of aHUS and, consequently, a lack of understanding. For example, a relative (R^2^) mentioned: “During the period that (name patient) was ill, I had no friends at all. I was unable to connect with anyone. I didn’t have time for it at all.” In addition, effective support was often restricted by the inability of others to fully comprehend the experiences of patients with aHUS and their families: “Almost nobody understands the feeling. That makes it very hard, but that also makes it very lonely” (R^2^). Furthermore, 2 grandparents and 1 parent mentioned: “They (outsiders) just don’t know what aHUS includes. It is a disease that can relapse at any time. He (patient) will have this disease lifelong. Even though it isn’t visible, he still has it” (R^6^ and R^7^) and “maybe I would just like to keep reminding outsiders that it’s just (still) there. We still have to go to the hospital on a regular basis” (R^4^). Particularly, the unpredictability of aHUS made it difficult to connect: “That uncertainty, that is difficult for those around you to feel. Of course, that is something you particularly feel yourself. The better your child is feeling, the more the outside world thinks: well, it's all right there. However, that uncertainty will never go away” (R^1^). Finally, a patient concluded: “To the outside world, it is easier to have a broken limb” (P^4^).

#### Professional Psychosociological Support

Implementing professional psychosociological support for relatives, particularly spouses and children of patients, throughout all stages of the disease, is needed. For example, a patient (P^1^) noticed: “I think that, in general, there is often-and this is not just for aHUS but also other conditions-too little attention paid to the partner or immediate relatives of a patient.” In addition, a relative (R^3^) stated: “I would have appreciated it if the nephrologist would have (actively) referred us to a (psycho)social worker. (To evaluate) whether they could support us.” Later, she added: “I needed care as well.”

#### Peer Support

Although interest in peer support varied, those who did engage with it appreciated it: “I have experienced the live events (aHUS peer meeting days) as very valuable” (R^3^). In addition, active referral to the patient association has been opted by both patients and relatives: “They should offer spouses the option to talk to peers” (R^2^) and “doctor needs to refer patients to the patient association” (P^1^).

### Information Accessibility and Clarity

The need for improved information was emphasized most frequently and consistently throughout all interviews. Better knowledge of aHUS was considered to positively affect all themes, particularly control and support. Participants felt that current information and its sources are insufficient, and they cited the complexity of the disease as the major challenge: “Current information is insufficient. We have no idea where to obtain this information” (R^6^ and R^7^) and “I think the doctor is 100%, how she explained it and everything. Only then it is still difficult to understand, because we are not doctors” (R^9^) and “you don’t have the energy to search for it (information), because you’ve got enough to worry about already. Doctors should take the time to explain it to you” (R^3^). Although participants often relied on doctors for information, many felt even their knowledge was lacking: “They (peripheral hospital) didn’t know aHUS that well, so they couldn’t provide us with the right information (in the acute phase)” (R^8^). Furthermore, information they did receive was too difficult or extensive and the need for simplified information in the Dutch language, an easy-to-understand format, and in an accessible place was suggested: “I just don’t understand it (information of aHUS on the internet). It is way too complicated. It would be great if there was something in easy language” (R^4^). One spouse (R^2^) added: “It would help me to have just 1 page of information. That would be manageable.” Two grandparents (R^6^ and R^7^) stated: “I would like a website with information about what aHUS entails and what are the consequences. But also how others experienced aHUS.” In addition, information on carriership, pregnancy, and the emotional impact of transplantation in relation to aHUS was desired.

### Challenges in Medical Care

#### Negative Experiences and Desires

A doctors’ lack of expertise also led to an inability to correctly assess the severity of the disease. This could delay (relapse) diagnosis: “If the GP or the peripheral hospital would have known aHUS symptoms a bit more, I think we would have been able to treat it a little sooner. The annoying thing about a rare disease is that, as a doctor, you cannot know everything” (R^1^) and “Our experience (which led to kidney transplant loss due to a delay in relapse diagnosis) of not being heard, believed, or taken serious is just so traumatic” (R^2^). Due to the infrequency of aHUS, early and selfless referral to expertise centers is needed: “I had to explicitly request a referral to the expertise center myself” (P^4^). and “We were just really lucky that we had a pediatrician who didn’t think: 'I’m going to score on this patient myself’ ” (R^1^). Finally, personalizing care was found to be important; however, some participants also requested a uniform protocol across (academic) hospitals: “Patients already have enough insecurities. Please make it clear to just have 1 protocol, which everyone follows” (P^4^).

#### Positive Experiences

On the contrary, many positive experiences with medical care were shared: “They (doctors) really gave me confidence that I could just call-in case of doubt. If I call now, we can come in immediately. I think that’s very valuable. I am glad that they (doctors) were able to give me that feeling. I never feel like a number there, which is nice” (R^4^). A patient (P^1^) adds: “What I expect from a doctor is knowledge, but also not treating a file but a patient.” Participants also praised (the need for) teamwork in managing aHUS: “We (doctor and parent) are equal. We both have a role in this game” (R^1^).

### Complexities of Genetic Testing and Carriership

The majority of the participants intentionally did not perform genetic testing in family members. One spouse (R^2^) about testing her children: “Because if you know, what do you know? What’s the benefit? Also look at the disadvantages. What can you do with that information (of being a carrier).” In addition, insurances were mentioned as a reason to refrain from testing. One participant (R^10^) is a known carrier, but she stated that “aHUS does not influence her life” other than some extra alertness at times of infection.

## Discussion

This was the first study to obtain in-depth insights into the experiences, needs, and desires of 19 Dutch patients with aHUS and their relatives through interviews, while including the IPH model as a guide. Participants had a wide variety of medical history, including disease severity, need for kidney transplantation, and eculizumab, or plasma therapy treatment, which led to interpersonal differences in disease experience. Nevertheless, this study revealed that aHUS has significant long-term impact on various domains.

Adult patients frequently reported permanent restrictions in bodily function. Particularly, fatigue hindered their ability to carry out daily activities, including paid work. Similar to our findings, 76% of patients with aHUS previously expressed a need for professional changes, whereas fatigue and concentration or memory problems have been identified as common long-term outcomes of thrombotic microangiopathy.^13^, ^14^, ^15^ In our study, emotional distress affected both patients and their relatives, throughout all phases of the disease. Although limited, data on psychological outcomes of aHUS indicated high levels of anxiety in 44% of patients and overall stress symptoms in almost all patients (97%) and relatives.^16^^,^^17^ A study by Azoulay *et al.*^13^ confirmed these findings, reporting high rates of mental health symptoms, posttraumatic stress disorder, and quality of life alterations in patients with aHUS up to years after intensive care unit discharge.

In our study, patients felt that there was attention paid to their mental well-being, to some extent by medical professionals but primarily by their support system. However, it was found remarkable that the emotional burden of those central to their support system, particularly spouses, parents, and children, was often severe but neglected. Long-term emotional and psychological distress that significantly disrupts family and daily life has been previously reported by relatives of patients with HUS.^18^ Posttraumatic stress symptoms may last for >15 years post-HUS, and in cases of chronic kidney disease, relatives may take on various responsibilities that lead to (additional) emotional burden and impacts family dynamics.^16^^,^^19^ The total economic burden of posttraumatic stress due to utility losses is significant, and developing preventive health care strategies aimed at addressing these issues are deemed necessary to further improve outcomes and cost-effectiveness of aHUS treatment.^13^^,^^16^^,^^20^ Our study highlights the need for professional psychosociological support, especially for relatives, throughout all stages of the disease. Extra support through active referral to patient associations and peer support were also suggested. As demonstrated in another study, social media groups may also be of support to patients with rare diseases.^21^

In aHUS, the potential relapsing nature and unpredictability can contribute to a sense of loss of control. Although eculizumab treatment is known to improve quality of life, discontinuation-standard of care for Dutch patients with aHUS does not have a long-lasting impact on quality of life.^4^^,^^9^^,^^14^ Furthermore, neither the occurrence nor the number of relapses is related to long-term mental health.^13^ Our study found that participants gained confidence after a period of time, especially after experiencing multiple potential triggering events without a relapse. Both patients and relatives emphasized the importance of increased understanding of the disease mechanism to regain control. Empowering autonomy and disease ownership has been previously described in the self-management of patients with glomerular diseases.^22^ However, this can be hindered by difficulties in interpreting the reliability of information, the continuously evolving understanding of disease mechanisms, and the unpredictability of a disease course.^22^, ^23^, ^24^ In addition, our study found that current information about aHUS was inadequate and overly complex, with many patients relying on doctors for information but finding their knowledge and ability to correctly assess the severity of the disease lacking. Referral to expertise centers and increased accessibility to easy-to-understand information in the Dutch language were opted as essential improvements in aHUS care. Finally, despite facing numerous challenges, patients and relatives also reported positive experiences with the medical care they received. Most participants eventually found acceptance and an increased appreciation for life and the caring people around them.

This study had some limitations. First, we did not report time from disease onset to interview or a medical history of relapse(s), which may have introduced time-lapse bias. Second, our interviews covered experiences with aHUS episodes both before and after the introduction of eculizumab, which is known to have affected kidney-related outcomes and quality of life significantly. Although we used purposive sampling of some participants, the pre-eculizumab era may have had a more pronounced effect on our results, particularly regarding long-term bodily functions and emotional impact. Third, despite our sampling method, we cannot exclude the possibility that participants with more negative experiences were more willing to participate. Fourth, we assigned 3 interviewers to ensure geographical coverage. Although we provided extensive training and a topic-guide, a single interviewer could have further improved interview consistency. Fifth, 2 authors, 1 of whom was an interviewer, had personal experiences with aHUS. Although they were not directly involved in the analysis, we cannot completely rule out their experiences or assumptions influencing the study results. Sixth, although data of 1-to-1 and family setting interviews appeared to be comparable (showing similar themes and discussion of sensitive topics) and a comfortable, nonjudgmental environment was pursued at all times, we cannot completely exclude response bias due to social dynamics. Seventh, only participants aged >15 years were initially considered eligible to participate. Consequently, the majority of children’s perspectives were obtained via their parents. Finally, we predominantly included male patients. Despite the reach of data saturation, we cannot exclude that inclusion of more female patients could have contributed to additional themes (e.g. pregnancy-related).

In conclusion, this unique study obtained insights into the experiences, needs, and desires of patients with aHUS and their relatives. Shortcomings in aHUS health care, especially regarding support and information provision, were identified. Future aHUS health care should cover the broad perception of health and expand its focus to the psychosociological aspects of the disease. Although future research should target specific subgroups within patients with aHUS and families, for now, the perspectives obtained in this study lay the foundation for the development of innovative and application of existing personalized (support) interventions. Finally, the holistic approach of our study and majority of the insights could be broadly applicable to other rare (kidney) diseases.

## Disclosure

All the authors declared no competing interests.

## Supplementary Material

Supplementary File (PDF) **Table S1.** Consolidated criteria for reporting qualitative studies (COREQ): 32-item checklist. **Topic Guide Interview and Evaluation**

## Footnotes

Supplementary File (PDF)
**Table S1.** Consolidated criteria for reporting qualitative studies (COREQ): 32-item checklist.
**Topic Guide Interview and Evaluation**
