# Supplementary material for: Living with Atypical Hemolytic Uremic Syndrome in the Netherlands: Patient and Family Perspective
Source: Kidney Int Rep. 2024 Apr 27;9(7):2189–97. doi: 10.1016/j.ekir.2024.04.047 (PMC11284443; doi:10.1016/j.ekir.2024.04.047)
Supplement: Supplementary File (PDF) — Table S1. Consolidated criteria for reporting qualitative studies (COREQ): 32-item checklist. Topic Guide Interview and Evaluation [file mmc1.pdf]

Supplementary Material

Topic Guide and Evaluation

Topic Guide Interview

Topic Guide Interview Eigen Regie

**Participant nr. :**		**Treatment patient:**	☐ aHUS no kidney transplantation<br>☐ aHUS after kidney transplantation
**Age :**		**Patient on ECU :**	☐ Yes ☐ No
**Sex :**	☐ Male ☐ Female ☐ other	**ECU schedule :**	☐ Standard dosing<br>☐ Prolonged interval ☐ ECU discontinued
**Relation to pat :**			
**Date :**		**Interview by :**	☐ 1 ☐ 2 ☐ 3

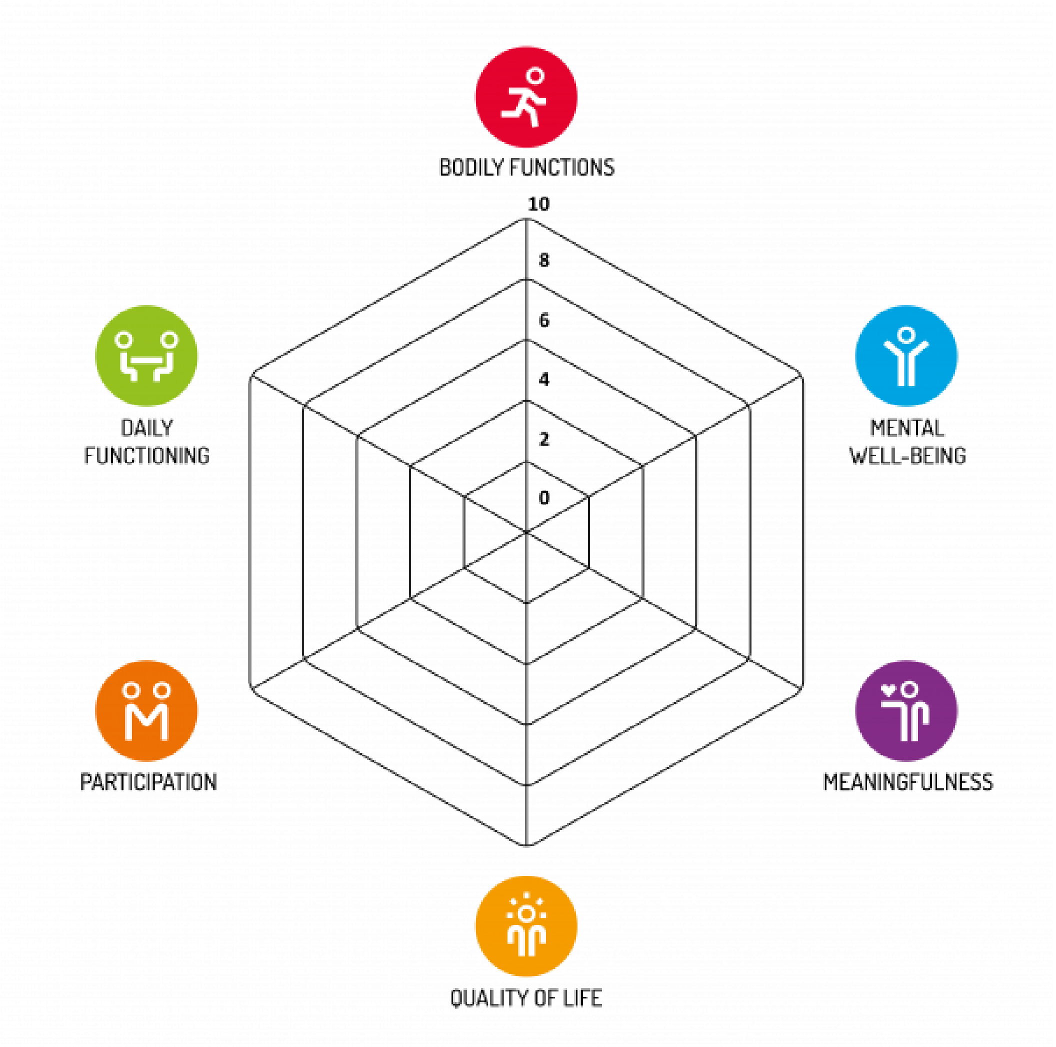

IPH model

**CURRENT AND DESIRED SITUATION**	In example:
- **Fill in IPH model for current situation**<br>- Let participants choose the for them most important themes in the IPH model. Discuss according to these themes:<br>○ **Current situation**<br>○ **Desired situation** (needs and desires)<br>○ **Factors of influence** (both positive and negative)<br>▪ **Inhibitory factors** *(what is stopping him/her to reach the desired situation?)*<br>• Personal<br>• External (others, resources or situation)<br>▪ **Stimulating and necessary factors** *(What is currently experienced as positive? What is needed to achieve the desired situation?)*<br>• Personal<br>• External (others, resources or situation)	*How are you? Where do you stand? In which area do you want to see growth? How can that growth be achieved? What can you do yourself to get there? What do you need from others for that growth? What is achieved or different if the desires situation is there? What situation does the participant come from? What growth has already been achieved?*
**PREVIOUS SITUATION (history)**	In example:
- **Let the participant tell about the 'acute' phase.** *This is the phase in which medically (traumatic) events occurred, for example: period in which he/she or a family member was diagnosed with aHUS, period of (first) hospitalization, period of recurrence of aHUS, other traumatic (medical) events to the participant.*<br>- Discuss further:<br>○ **Unpleasant factors** (personal, others, resources or situation)<br>○ **Valued factors** (personal, others, resources or situation)<br>○ **Desired factors or situation**<br>▪ Were there factors already realized by themselves at that time?	*What would have been necessary to have achieved the desired situation at the time? What factors (example: actions, medical personnel, information) were valued or desired during that period?*
**OTHER INFORMATION (considering the interview)**	AFTER interview, fill in:

Topic Guide Evaluation Eigen Regie

Radboudumc — nvn nierpatiënten vereniging nederland

**Participant nr. :**		**Date interview :**	
**Date :**		**Interview by :**	☐ 1 ☐ 2 ☐ 3

**EVALUATION INTERVIEW**	
**Experiences interview**	Discuss
**Personal**	e.g. emotions
**ADDITIONAL**	
**Short summary and evaluation of interview**	Provide
**Additional (new) topics?**	Discuss
**Desires**	DO NOT conduct a completely (new) interview. Evaluate only to discuss previously mentioned important factors and/or additional factors.
Inhibitory factors	
Stimulating factors	
Necessary factors	
**Medical history**	
Desires factors/situation	
**OTHER INFORMATION**	

Supplementary Table S1 – *Consolidated criteria for reporting qualitative studies (COREQ): 32-item checklist*

**No. Item**	**Guide questions/description**	**Reported on Page # (lines)**
**Domain 1: Research team and reflexivity**		
Personal Characteristics		
1. Inter viewer/facilitator	Which author's conducted the interview or focus group?	5 (99-101, 118)
2. Credentials	What were the researcher's credentials? E.g. PhD, MD	5 (116-119)
3. Occupation	What was their occupation at the time of the study?	5 (116-119)
4. Gender	Was the researcher male or female?	NA
5. Experience and training	What experience or training did the researcher have?	5 (99-101, 116-117)
Relationship with participants		
6. Relationship established	Was a relationship established prior to study commencement?	5 (101)
7. Participant knowledge of the interviewer	What did the participants know about the researcher? e.g. personal goals, reasons for doing the research	4 (75-76)
8. Interviewer characteristics	What characteristics were reported about the inter viewer/facilitator? e.g. Bias, assumptions, reasons and interests in the research topic	5 (116-119)<br>12 (356-358)

No. Item	Guide questions/description	Reported on Page # (lines)
**Domain 2: study design**		
Theoretical framework		
9. Methodological orientation and Theory	What methodological orientation was stated to underpin the study? e.g. grounded theory, discourse analysis, ethnography, phenomenology, content analysis	4 (63)<br>5 (109-116, 119-121)
Participant selection		
10. Sampling	How were participants selected? e.g. purposive, convenience, consecutive, snowball	4 (73-87)
11. Method of approach	How were participants approached? e.g. face-to-face, telephone, mail, email	4 (74)
12. Sample size	How many participants were in the study?	4 (64)<br>5 (129-131)
13. Non-participation	How many people refused to participate or dropped out? Reasons?	NA
Setting		
14. Setting of data collection	Where was the data collected? e.g. home, clinic, workplace	5 (101-106)
15. Presence of non-participants	Was anyone else present besides the participants and researchers?	4 (82-85)
16. Description of sample	What are the important characteristics of the sample? e.g. demographic data, date	4 (64-65)<br>5 (131)<br>12 (363-365)<br>16 (Table 1)
Data collection		
17. Interview guide	Were questions, prompts, guides provided by the authors? Was it pilot tested?	4 (65, 91-97)<br>5 (99-100)<br>Supplementary file A
18. Repeat interviews	Were repeat inter views carried out? If yes, how many?	NA
19. Audio/visual recording	Did the research use audio or visual recording to collect the data?	5 (106)
20. Field notes	Were field notes made during and/or after the inter view or focus group?	5 (123-124)
21. Duration	What was the duration of the inter views or focus group?	5 (104)<br>6 (132)
22. Data saturation	Was data saturation discussed?	5 (115)
23. Transcripts returned	Were transcripts returned to participants for comment and/or correction?	5 (105-106)
**Domain 3: analysis and findings**		

Data analysis		
24. Number of data coders	How many data coders coded the data?	5 (110-113)
25. Description of the coding tree	Did authors provide a description of the coding tree?	5 (109-115)<br>16 (Table 2)
26. Derivation of themes	Were themes identified in advance or derived from the data?	5 (119-120)
27. Software	What software, if applicable, was used to manage the data?	5 (113-114)
28. Participant checking	Did participants provide feedback on the findings?	5 (105-106)
Reporting		
29. Quotations presented	Were participant quotations presented to illustrate the themes/findings? Was each quotation identified? e.g. participant number	5 (124-125)
30. Data and findings consistent	Was there consistency between the data presented and the findings?	6-10 (quotes provided)
31. Clarity of major themes	Were major themes clearly presented in the findings?	16 (Table 2)
32. Clarity of minor themes	Is there a description of diverse cases or discussion of minor themes?	16 (Table 2)
